# The Curcumin Analogue, MS13 (1,5-Bis(4-hydroxy-3- methoxyphenyl)-1,4-pentadiene-3-one), Inhibits Cell Proliferation and Induces Apoptosis in Primary and Metastatic Human Colon Cancer Cells

**DOI:** 10.3390/molecules25173798

**Published:** 2020-08-20

**Authors:** Nor Isnida Ismail, Iekhsan Othman, Faridah Abas, Nordin H. Lajis, Rakesh Naidu

**Affiliations:** 1 Jeffrey Cheah School of Medicine and Health Sciences, Monash University Malaysia, Jalan Lagoon Selatan, Bandar Sunway 47500, Selangor, Malaysia; isnidai@gmail.com (N.I.I.); iekhsan.othman@monash.edu (I.O.); 2 UniKL MESTECH, A1-1 Jalan TKS1, Taman Kajang Sentral, Kajang 43000, Malaysia; 3 Laboratory of Natural Products, Faculty of Science, University Putra Malaysia, Serdang 43400, Malaysia; faridah@food.upm.edu.my (F.A.); nordinlajis@gmail.com (N.H.L.); 4 Department of Food Science, Faculty of Food Science and Technology, University Putra Malaysia, Serdang 43400, Malaysia

**Keywords:** curcumin analogue, diarylpentanoid (DAP), colon cancer, cell proliferation, apoptosis, 1,5-bis(4-hydroxy-3-methoxyphenyl)-1,4-pentadiene-3-one, Bcl-2, Caspase-3

## Abstract

The cytotoxic and apoptotic effects of turmeric (Curcuma longa) on colon cancer have been well documented but specific structural modifications of curcumin have been shown to possess greater growth-suppressive potential on colon cancer than curcumin. Therefore, the aim of this study is to identify the anti-cancer properties of curcumin analogue-MS13, a diarylpentanoid on the cytotoxicity, anti-proliferative and apoptotic activity of primary (SW480) and metastatic (SW620) human colon cancer cells. A cell viability assay showed that MS13 has greater cytotoxicity effect on SW480 (EC_50_: 7.5 ± 2.8 µM) and SW620 (EC_50_: 5.7 ± 2.4 µM) compared to curcumin (SW480, EC_50_: 30.6 ± 1.4 µM) and SW620, EC_50_: 26.8 ± 2.1 µM). Treatment with MS13 at two different doses 1X EC_50_ and 2X EC_50_ suppressed the colon cancer cells growth with lower cytotoxicity against normal cells. A greater anti-proliferative effect was also observed in MS13 treated colon cancer cells compared to curcumin at 48 and 72 h. Subsequent analysis on the induction of apoptosis showed that MS13 treated cells exhibited morphological features associated with apoptosis. The findings are also consistent with cellular apoptotic activities shown by increased caspase-3 activity and decreased Bcl-2 protein level in both colon cancer cell lines. In conclusion, MS13 able to suppress colon cancer cell growth by inhibiting cell proliferation and induce apoptosis in primary and metastatic human colon cancer cells.

## 1. Introduction 

Colorectal cancer (CRC) currently ranks as the third most prevalent cancer worldwide after lung and breast cancer [1]. CRC incidence was more common in Western countries [2,3] and raising drastically in some countries in the Asia-pacific regions [4]. Most CRC begins with the silencing of the *APC* gene which function includes organization of the cytoskeleton, modulation of cell migration, cell cycle and apoptosis regulation, and plays an important role in signal transduction of the Wnt-signaling pathway. It is involved in a multistep process of mutations causing gene silencing of the tumor suppressor (*APC*, *TP53*, and *SMAD4/DC4*) and mismatch repair (*hMSH2, hMLH1*, *hPMS1*, and *hMSH6*) genes and also gain of function of the oncogenes (*KRAS*, *PI3KCA*, and *BRAF*) [5]. The truncated proteins of these critical genes lead to the impairment of apoptosis, enhancement of cellular proliferation and defects in cell cycle control mechanism of the epithelial cells (colonocytes) that lining the bowel, which usually leads to the formation of malignant adenomatous polyps as observed in 96% of CRC cases. Later, the malignant polyps may invade, protrude into the bowel wall, metastasize via the lymph and circulatory systems, and finally cause fatality. 

Regardless of the advancement in CRC treatment such as early diagnosis, surgical resections, chemotherapy, and radiotherapy, the survival and recurrence rate of CRC patients have not improved. Instead, CRC has been identified as the second leading cause of cancer death worldwide with an estimation of more than 1.1 million death and 2.2 million new cases by year 2030. Moreover, chemotherapy and radiotherapy treatment are often accompanied with toxicity and serious side effects, thus affecting the quality of CRC patient’s life. Resistance towards multi-chemo drugs increased the recurrence rate and lower the 5-year chances of survival. Almost half of the patients receiving FOLFOX (Leucovorin + 5-FU + Oxaliplatin) were reported to develop chemo-resistance at a later stage of the chemo-treatment, contributing to the high incidence rate of cancer recurrence and metastasis to other organs [6,7]. The standard regimen for treating CRC, however, includes CAPOX (capecitabine + oxaliplatin), FOLFIRI (Leucovorin +5-FU + Irinotecan), and FOLFOX [8,9]. However, until recently precision medicine which incorporates specific therapeutic agents which correlates with specific tumor characteristics that lead to abnormal expression and defect in biological pathways have gained much interest in the clinical trial setting as well as development of drugs for CRC [10]. The molecular targets and clinical trials of CRC include targeting the EGF/EGFR, VEGF-VEGF Receptor (VEGFR), MAPK-RAS/FAR/MEK/ERK and PI3K-mTOR pathways via the usage of anti-EGFR and anti-angiogenic monoclonal antibodies, tyrosine kinase inhibitors (TKIs) and immune checkpoint inhibitors (ICI) [11,12,13,14]. 

As such, several studies have focused on the discovery of new therapeutic approaches for CRC from compounds of natural sources with fewer side effects that target these pathways, synergistically improve chemo-drug efficacy and are more effective. Curcumin, an active compound derived from the rhizomes of the turmeric plant (*Curcuma longa*) has gained much attention as an anti-cancer agent with minimal side effects on CRC. It has the ability to regulate multiple signaling pathways, including cell cycle via down-regulation of cyclin D1, CDk4, and p53; Wnt signaling by up-regulating GSK3b and down-regulating E-cadherin, β-catenin, and c-MYC, restricting angiogenesis (NF-KB, AP-1, iNOS, NO, *5-LOX*, *COX-2*, *MMP*s), up-regulating miR-15 and miR-16 and promotes genomic stability via down-regulation of DNA methyl transferase 1 and histone protein alteration [15,16]. Notwithstanding its anti-cancer properties, clinical trial and pharmacokinetic data on curcumin displayed poor absorption and bio distribution, low bioavailability [17,18,19], inadequate biological stability [20], poor aqueous solubility [20,21], rapid metabolism [22], and rapid systemic elimination [19,23]. Curcumin which is hydrophobic [20] undergone biotransformation into curcumin glucuronides in the intestine and liver [24,25], which it is rapidly metabolized [22] and eliminate via feces [20,23,26].

However, previously, a variety of novel analogues of curcumin have been developed by replacing or introducing different functional moieties-biaryl rings, ^®^-diketone and diene chain [27] at different positions of its structure [19]. Modification of curcumin basic structure that comprises two phenol groups connected by two α, β unsaturated carbonyl group leads to a range of different curcumin analogues with improved pharmacokinetic properties, biological and anti-cancer activities [27,28,29,30,31,32,33,34,35,36]. Several series of newly synthesized curcumin analogues of diarylpentanoid (DAP) displayed highly improved anti-cancer activity compared to curcumin. The DAPs, which possess two aromatic rings (aryl groups), were joined together by five carbon chains have been shown to suppress cancer growth via modulation and regulation of NFKB [37,38], STAT3 [39,40], AKT-PTEN [41], MAPK-ERK [38], cell cycle arrest, and apoptosis [41]. The anti-cancer therapeutic properties of DAPs have been studied in several cancer cell lines such as colorectal (GO-Y035, GO-Y030, FLLL-11, FLLL-12, HO-3867, and EF24), breast (GO-Y035, GO-Y030, HO-3867, EF24, and EF31), lung (GO-Y035, HO-3867, and EF24), liver bile duct (GO-Y030), pancreatic (GO-Y035, GO-Y030, FLLL-11, FLLL-12, and EF31), prostate (HO-3867, EF24, and ca27) [42], thyroid gland (GO-Y035 and GO-Y030), stomach (GO-Y035) ovarian (HO-3867, EF24, and EF31) and cervical (EF24) cancers as summarized by Paulraj et al., 2019 [43].

**Figure 1:**
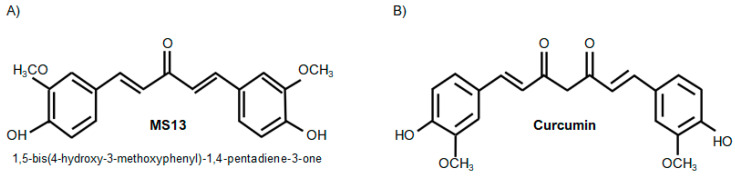
Molecular structure of MS13 (**A**) and curcumin (**B**).

1,5-Bis(4-hydroxy-3-methoxyphenyl)-1,4-pentadiene-3-one, a DAP synthesized by modification of acetone carbon linker, aryl rings and removal of β-diketone moiety (Figure 1A) demonstrated improved in vivo pharmacokinetic profiles than curcumin (Figure 1B) with increased half-life, better absorption, and low metabolism [44,45]. Accumulating evidence demonstrated that this DAP (Figure 1A) which contain 3′OCH_3_-4′OH had stronger cytotoxicity activity compared to curcumin (Figure 1B) against non-small cell lung cancer-NCI-460, melanoma-UACC-62, ovarian cancer-OVCAR-3, renal cancer-786-0, prostate cancer-PC3 [42,46,47], LNCap [42] and DU145 [47], breast cancer-MDA-MB231 [42,48] and MCF-7 [42,46], cervical cancer-HeLa and CaSki [49], nasopharyngeal cancer-CNE [44,50], leukemia-K-562 [46] and colon cancer HT-29 [46], HCT116 and SW480 [45]. However, studies involving DAPs on colon cancer SW480 which bearing chromosomal instability (CIN), KRAS and p53 mutations were limited to the cytotoxicity effect, while the anti-proliferative and apoptosis were not performed. Hence, it is interesting to evaluate the anti-cancer potential properties of the 1,4-pentadiene-3-one on colon cancer cells carrying KRAS mutation and exhibit molecular phenotypes of chromosomal instability (CIN) [51] that accounts 85% of all sporadic CRCs [52,53]. Therefore the aim of the present study was to determine the cytotoxicity, anti-proliferative and apoptotic activity of 1,5-bis(4-hydroxy-3- methoxyphenyl)-1, 4-pentadiene-3-one which is MS13 on human primary (SW480) and metastatic (SW620) colon cancer cells carrying KRAS and p53 mutations and also undergone CIN. 

## 2. Results

### 2.1. MS13 and Curcumin Reduce Colon Cancer Cell Viability at Differential Doses 

**Figure 2:**
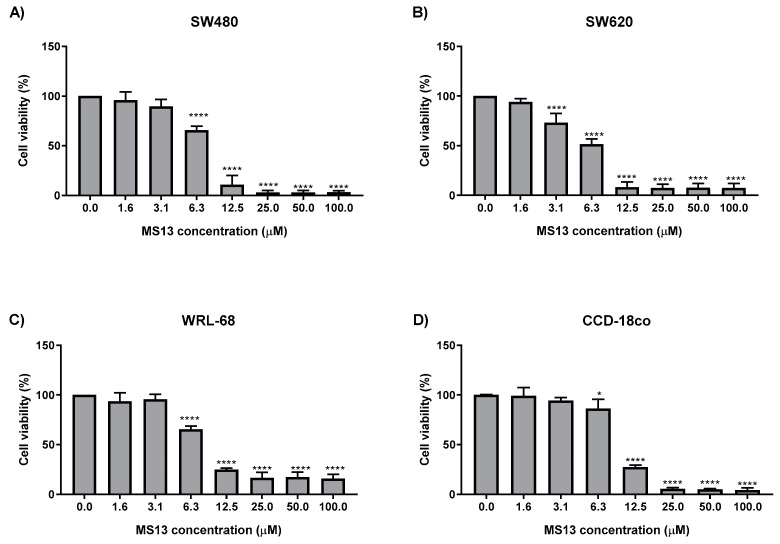
The cytotoxicity effect of MS13 on (**A**) SW480, (**B**) SW620, (**C**) WRL-68 and (**D**) CCD-18co cell viability. Experiments were performed in triplicates and results were compared between three independent experiments. Statistically significant differences between the means of values obtained with treated vs untreated are represented by * for *p* ≤ 0.05, ** for *p* ≤ 0.01, *** for *p* ≤ 0.001 and *p* **** for *p* ≤ 0.0001. Error bars indicate mean ± SEM.

MS13 showed dose-dependent cytotoxicity effect in cancer (SW480 and SW620) and normal (WRL-68 and CCD-18co) cells when incubated for 72 h (Figure 2). Untreated cells served as control and their viability was set as 100%. MS13 able to demonstrate cytotoxic effect in both cancer cell lines with a significant decrease in cell viability at 6.3 µM onwards in SW480 (Figure 2A) and 3.1 µM onwards in SW620 cells (Figure 2B). However, for the normal cells MS13 displayed a significant decreased in cell viability at doses of 6.3 µM onwards in WRL-68 and CCD-18co with cell viability approximately of 65% and 86%, respectively. MS13 showed inhibition of SW480 cell growth with cell viability decreasing to 66% at 6.3 µM and gradually to 13% at 12.5 µM and less than 3% at 25 µM onwards when compared to control. Cytotoxicity effect of MS13 towards SW620 followed a similar trend by exhibiting a significant decrease in cell growth beginning from 3.1 µM (cell viability: 73%) followed by a 49% reduction of cell viability at 6.3 µM and decreasing to less than 9% at 12.5 µM onwards. 

**Figure 3:**
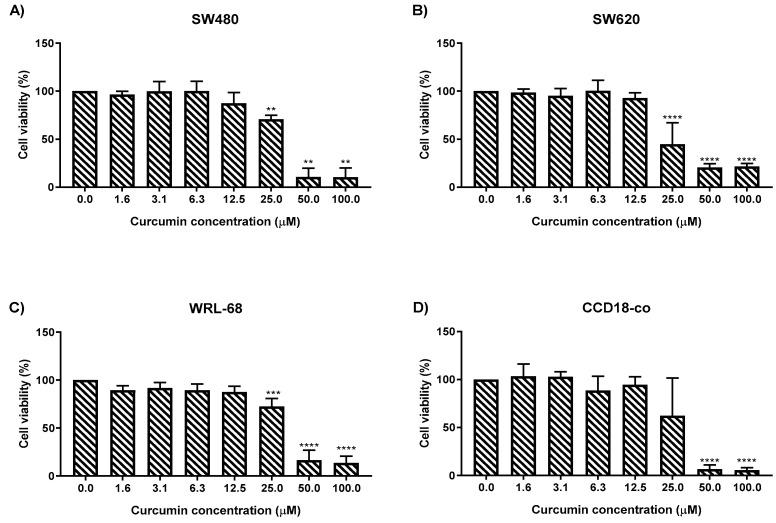
The cytotoxicity effect of curcumin on (**A**) SW480, (**B**) SW620, (**C**) WRL-68 and (**D**) CCD18-co cell viability. Experiments were performed in triplicate and results were compared between three independent experiments. Statistically significant differences between the means of values obtained with treated vs. untreated are represented by * for *p* ≤ 0.05, ** for *p* ≤ 0.01, *** for *p* ≤ 0.001 and *p* **** for *p* ≤ 0.0001. Error bars indicate mean ± SEM.

Curcumin treatment for 72 h also showed reduction in cell viability of SW480 and SW620 in a dose-dependent manner (Figure 3). Significant growth inhibition was observed with cell viability approximately 71% in SW480 (Figure 3A) and 45% in SW620 cells (Figure 3B) when treated with curcumin at the same dosage of 25 µM and higher. However, in normal cells a significant cell growth inhibition with cell viability of approximately 72% and 7% was observed in WRL-68 (Figure 3C) and CCD-18co (Figure 3D) following curcumin treatment at dosages of 25 µM and 50 µM respectively when compared to the untreated cells. 

Table 1 showed the values obtained from MS13 and curcumin treatment on colon cancer (SW480 and SW620) and normal (WRL-68 and CCD-18co) cells were based on the 72 h incubation period. The EC_50_ values of MS13 are shown in Table 1. Curcumin displayed higher EC_50_ values compared to MS13. The range of the EC_50_ of MS13 for all cell lines were less than 10 µM, while the EC_50_ values of curcumin were between 26 µM–31 µM. The cytotoxicity effects of all compounds were further evaluated for their toxicity against normal liver epithelial (WRL-68) and normal colon (CCD-18co) cell lines by calculating the *SX* value (Table 2). MS13 showed a high *SX* value that exceeds 100 in both cancer cell lines compared to curcumin. *SX* value that exceeds 100 indicates that MS13 cytotoxicity is greater in cancer cells compared to the normal cells.

### 2.2. MS13 and Curcumin Exhibit Anti-Proliferative Activity on Colon Cancer Cells 

**Figure 4:**
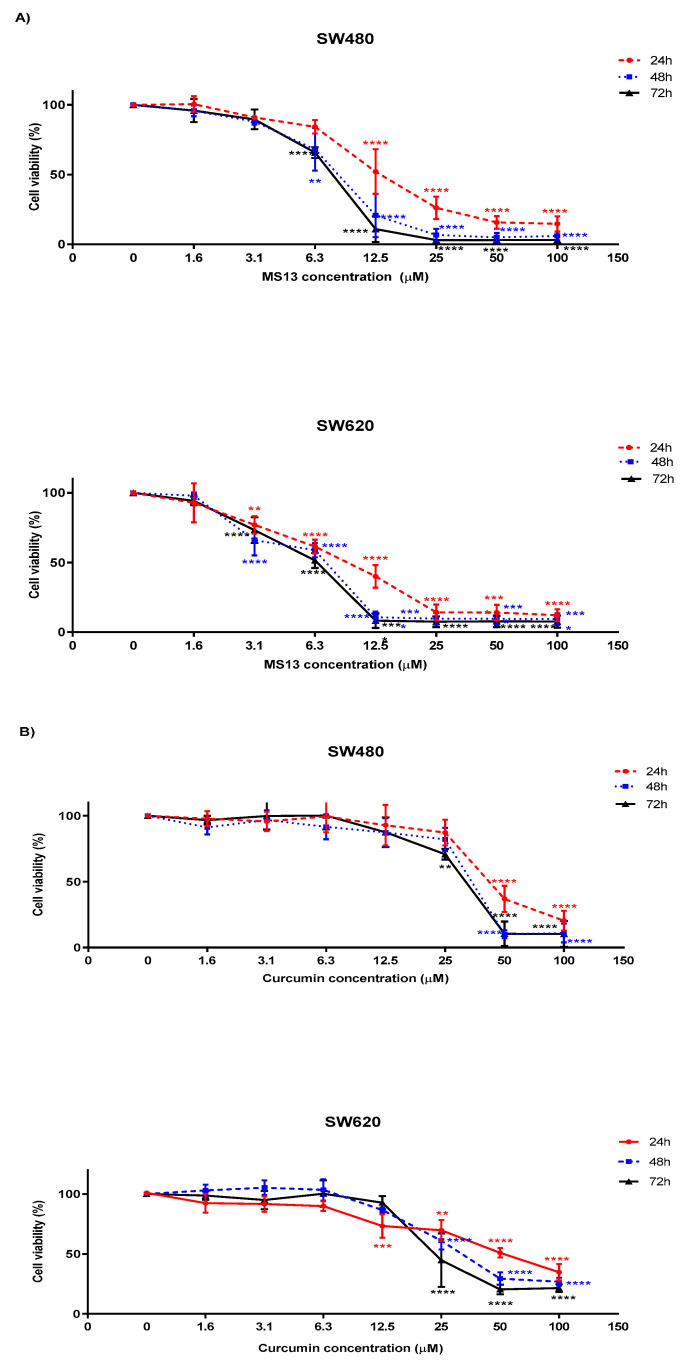
The anti-proliferative effects of (**A**) MS13 and (**B**) curcumin on SW480 and SW620 at 24, 48 and 72 h. Experiments were performed in triplicates and results were compared between three independent experiments. Asterisk indicates statistically significant (* for *p* ≤ 0.05, ** for *p* ≤ 0.01, *** for *p* ≤ 0.001 and **** for *p* ≤ 0.0001) differences between the means of values with treated vs untreated cells. Error bars indicate mean ± SEM.

Significant decline of SW480 cell viability was noted at 12.5 µM onwards for 24 h incubation period and 6.3 µM onwards for 48 and 72 hrs. MS13 treatment at 12.5 µM onwards, significantly reduce cell viability (Figure 4A) of SW480 cells by 48% at 24 h, and approximately 79% and 89% at 48 and 72 h respectively. Treatment by MS13 at the same dosage of 6.3 µM on SW480 cells for 48 and 72 h reduced cell viability to 68% and 66% respectively indicating inhibition of cell growth. Cell viability was observed less than 50% when treated with MS13 beginning at 25 µM for 24 h and 12.5 µM for 48 and 72 h when compared to the untreated cells. SW480 cells treated with 25 µM and higher for 24 h demonstrated a reduction of cell proliferation with cell viability approximately 26%, while treatment for 48 and 72 h at the same dosage caused cell proliferation to decline with cell viability approximately 7% and 3% respectively. MS13 also able to inhibit SW620 cell proliferation at a lower dosage compared to the SW480 cells. The cell proliferation was noted to decline beginning at 3.1 µM onwards following MS13 treatment for all time points when compared to the untreated. At this concentration, the reduction at 24 h was approximately 23%, and approximately 34% and 27% at 48 and 72 h compared to the untreated. Approximately cell viability at 62% (*p* ≤ 0.01) was observed upon 6.3 µM MS13 treatment for 24 h, followed by cell viability of 40% (*p* ≤ 0.0001) and 14% (*p* ≤ 0.0001) at 12.5 µM and 25 µM at the same time point. Cell viability of SW620 cells was reduced to less than 50% when treated with MS13 from dosage of 12.5 µM and above for 24, 48 and 72 h. Cell viability was noted approximately 14% at 24 h, less than 10% for 48 and 72 h upon MS13 treatment at 25 µM. Overall, treatment with MS13 resulted in a both dose- and time-dependent reduction in proliferative activity of SW480 and SW620 cells. In most cases, MS13 treatment for 48 and 72 h significantly caused higher anti-proliferative activity.

Curcumin on the other hand demonstrated moderate anti-proliferative activity compared to MS13 treatment on both cancer cell lines (Figure 4B). In SW480 cells, significant inhibition of cell viability was observed at 50 µM onwards for all time points upon treatment with curcumin. Cell viability was reduced to 37% at 24 h, 10% at 48 h and 11% at 72 h of the same dosage of curcumin treatment. Curcumin treatment below than 12.5 µM on SW480 cells showed no significant inhibition at all time points. A similar pattern of anti-proliferative activity was also observed upon curcumin treatment on SW620 cells. A higher dosage of curcumin is required to significantly reduce cell viability of the SW620 cells. Curcumin treatment on SW620 cells beginning from 25 µM onwards showed significant reduction of cell growth at all time points with approximately 30% for 24 h, 39% for 48 h and 55% for 72 h. Curcumin treatment on SW620 cells at 50 µM for 48 and 72 h caused significant reduction of cell viability by 70%.

### 2.3. MS13 Induces Apoptotic Morphological Changes as Observed by Annexin V/PI Double Staining

To evaluate induction of apoptosis in the colon cancer cells upon MS13 treatment, morphological changes related to cell death was first observed by examining the Annexin V-FITC/PI-stained colon cancer cells under the fluorescence microscope at EC_50_ and 2XEC_50_ of MS13 concentration for 24, 48 and 72 h. Annexin V binds to the cells that expressed phosphatidylserine (PS) on the cell surface and is found in apoptotic and necrotic cells, while PI is a membrane impermeant, unable to enter the intact cell membrane, which therefore only stained the late apoptotic and necrotic cells due to the lack of plasma membrane [54,55,56]. Hence, the cells were grouped into live cells which are invisible as they show no staining; necrotic (Annex-V-/PI+) are pale orange or red uniform nucleus; and the apoptotic cells which includes early and late apoptotic cells. Early apoptotic (Annex-V+/PI-) cells are either with green outer layer plasma membrane or stained green to yellow with loss of membrane integrity and chromatin condensation while late apoptotic (Annex-V+/PI+) cells are condensed or fragmented yellow-orange or orange chromatin [54,55,56]. 

**Figure 5:**
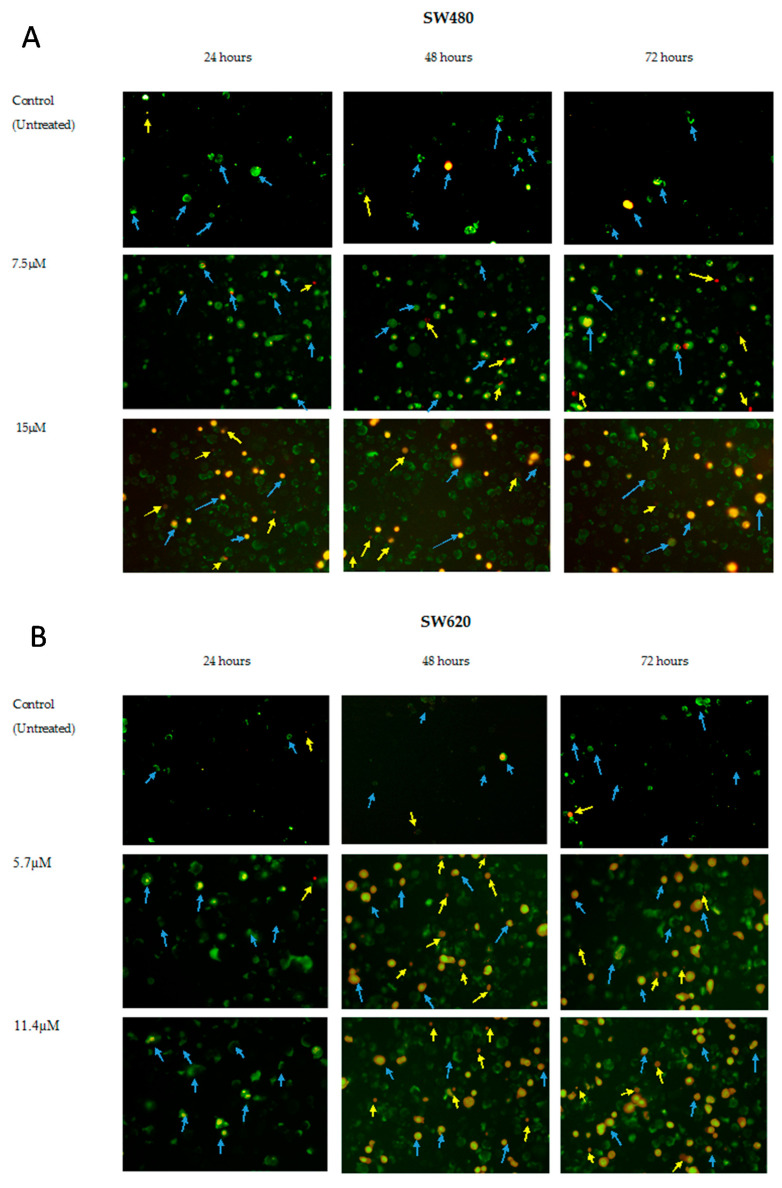
MS13 induces cell death as observed by apoptotic morphological changes in (**A**) SW480 (**B**) SW620 human colon cancer cells. Detection was by fluorescence microscopy of Annexin V/PI treated with MS13 for 24, 48 and 72 h at 200× magnification. Viable cells (Annexin V-/PI-) are invisible; Early apoptotic cells showed green plasma membrane (Annex-V+/PI-); Late apoptotic cells showed red staining throughout the nucleus with halo of green on plasma membrane indicating loss of membrane integrity (Annex-V+/PI+), chromatin condensation and fragmentation, stained green to yellow (Annex-V+/PI+), yellow-orange and orange (Annex-V+/PI+) (arrow in blue); necrotic cells (Annex-V-/PI+) showed uniformly red or pale orange (arrow in yellow).

In the Annexin-PI staining images (Figure 5A,B), there was an increased in the number of cells showing signs of apoptosis such as cells with green plasma membrane which represent early apoptosis, red staining throughout the nucleus with halo of green on plasma membrane indicating loss of membrane integrity, chromatin condensation and fragmentation, stained green to yellow, yellow-orange and orange which represent late apoptosis [56]. These observations were proportional to the increased MS13 doses and incubation time when compared to the untreated cells. Such changes were observed in response to the MS13 treatment in both SW480 (Figure 5A) and SW620 (Figure 5B). 

A large number of cells treated with MS13 were stained by Annexin-PI an indication of MS13 able to induce apoptosis. In SW480 cells (Figure 5A), treatment with 7.5 µM (EC_50_) of MS13 at 24, 48, and 72 h demonstrated a mix population of cells with green plasma membrane describing early apoptotic cells. SW480 cells treated with MS13 at higher dosage 15 µM (2XEC_50_) for 24, 48 and 72 h displayed increased mix population of cells stained with green, yellow, and orange indicating mixture of early and late apoptotic cells. Increased in number of apoptotic cells also observed as increased with incubation hours from 24 to 72 h at 7.5 µM and 15 µM of MS13. However, late apoptotic cells were observed as early as 24 h upon MS13 treatment at 15 µM, while necrotic cells represented by the pale orange and uniform red staining were mostly visible at 72 h upon MS13 treatment with 7.5 µM and 15 µM. 

In SW620 cells (Figure 5B), treatment with EC_50_ (5.7 µM) and 2XEC_50_ (11.4 µM) of MS13 at 24 h displayed a higher number of early apoptotic cells as seen by the increase of green and yellow stained cells and also cells with the green stained plasma membrane. There was an increase in late apoptosis cells following treatment with 5.7 µM and 11.4 µM MS13 at 48 and 72 h. The number of late apoptotic cells were higher with the increase of MS13 dosage and incubation time. Higher apoptotic cells were observed at 48 and 72 h compared to 24 h upon treatment with 5.7 µM and 11.4 µM. Noticeable pale orange and uniform red staining indicating necrosis was demonstrated higher in SW620 cells treated with MS13 at 48 and 72 h of higher dosage, 11.4 µM. Overall, morphological analysis demonstrated that treatment with MS13 increased number of apoptotic cells in SW480 and SW620 cells in dose- and time-dependent manner compared to untreated cells an indication of the cell death induction via apoptosis upon MS13 treatment for 24, 48, and 72 h. Necrotic cells however represented by the pale orange, and red staining were mostly visible at 72 h upon MS13 treatment at 5.7 µM and 11.4 µM. 

### 2.4. Quantification of Apoptotic and Necrotic Cells Induced by MS13

**Figure 6:**
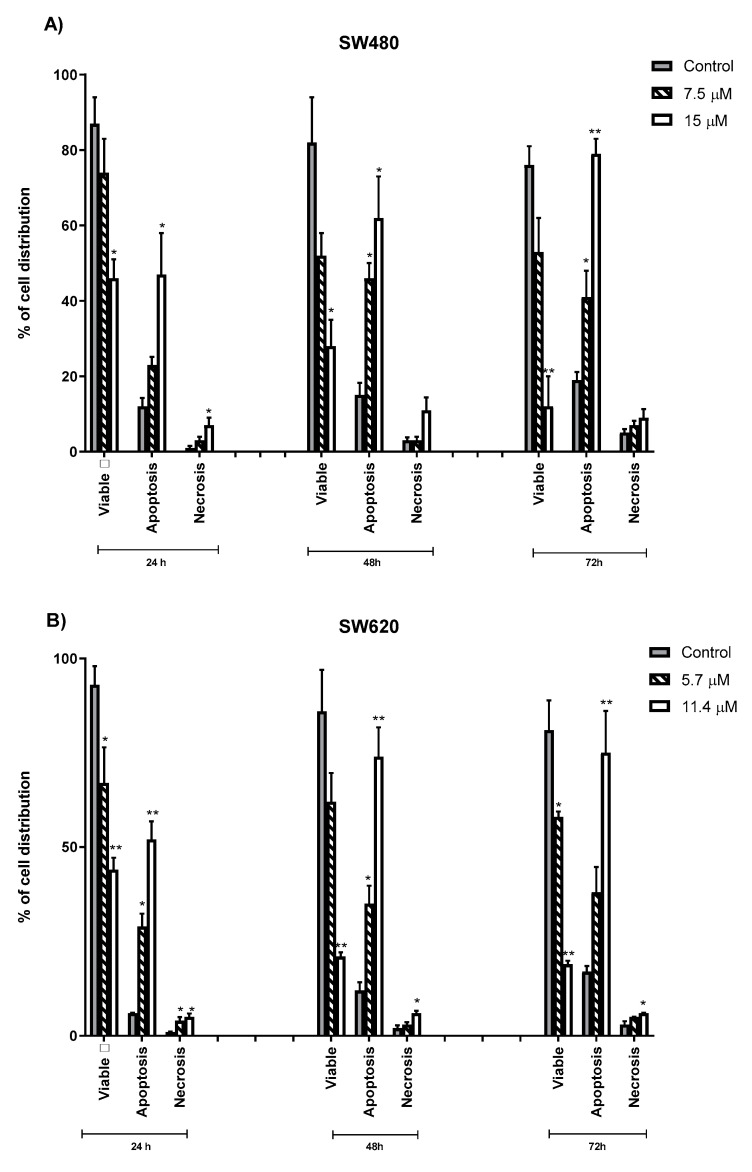
Percentage of viable, apoptotic, and necrotic cells in SW480 (**A**) and SW620 (**B**) treated with MS13 for 24, 48, and 72 h. Treated and non-treated cells were double-stained with Annexin V/ Propidium Iodide and a minimum of 200 cells were counted per sample. Experiments were performed in triplicates and results were compared between two independent experiments. Comparison between data sets was performed using ANOVA. Asterisk indicates statistically significant (* for *p* ≤ 0.05, ** for *p* ≤ 0.01, *** for *p* ≤ 0.001 and **** for *p* ≤ 0.0001) differences between the means of values with treated vs untreated cells. Error bars indicate mean ± SEM.

Figure 6 represents the quantification of cell death activity (%) associated with morphological changes by grouping the cells into viable, apoptotic (early+ late apoptotic) and necrotic cells on 5 randomly chosen microscopic fields (X40) of 200 cells. In both cell lines (SW480 and SW620), the percentage of viable cells decreased on exposure to longer incubation times. Increased apoptotic cells were noted in all dosage (EC_50_ and 2XEC_50_) of MS13 treatment at 24, 48, and 72 h compared to the untreated cells. In SW480 cells, MS13 treatment with 7.5 µM for 48 h significantly increased the apoptotic activity approximately up to 46% and reaching to 62% at 15 µM. SW480 treated cells with 15 µM for 72 h demonstrated the highest apoptotic activity (79%, *p* ≤ 0.01). Higher apoptotic activity was shown in SW480 cells upon MS13 treatment (7.5 µM and 15 µM) when incubated for 48 and 72 h compared to 24 h. Viable cells of the untreated was noted approximately more than 75% in all time points (24, 48, and 72 h). Interestingly, necrotic cells remain approximately less than 11% when treated with MS13 either at 7.5 µM or 15 µM at all time points. No significant difference of necrotic cells was also observed in SW480 cells between untreated and treated cells neither 7.5 µM nor 15 µM for 48 and 72 h. 

However, in SW620 cells, treatment of MS13 at dosage of 5.7 µM significantly induced approximately 25–35% of apoptotic cells, while at 11.4 µM more than 50% of cells demonstrated apoptosis when incubated for 24, 48 and 72 h, respectively. There is also an increase in apoptosis from 38% to 75% at 72 h following 5.7 µM and 11.4 µM MS13 treatment respectively. The apoptosis activity remains approximately 30% following treatment at 5.7 µM for 24, 48 and 72 h. Overall, MS13 able to induce apoptosis in both cell lines as shown by the morphological changes in a time- and dose-dependent manner.

### 2.5. MS13 Increases Caspase-3 Activity in Colon Cancer Cells

**Figure 7:**
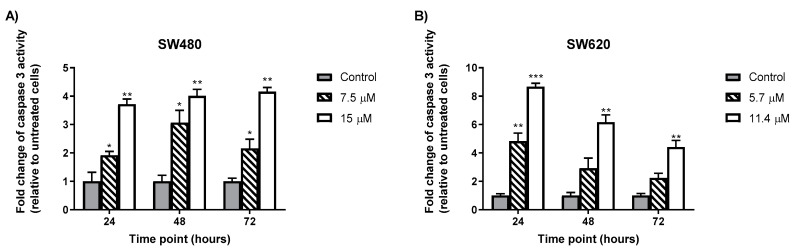
MS13 increases caspase-3 activity at different time points in SW480 (**A**) and SW620 (**B**) cells. Experiments were performed in duplicates and compared between two independent experiments. Asterisk indicate statistically significant (* for *p* ≤ 0.05, ** for *p* ≤ 0.01, *** for *p* ≤ 0.001 and **** for *p* ≤ 0.0001) differences between data sets for each treatment dose. Error bars indicate mean ± SEM.

Caspase-3 activity was increased at 24, 48, and 72 h in both colon cancer (SW480 and SW620) cells treated with MS13 at EC_50_ and 2XEC_50_ compared to the untreated cells (Figure 7). In SW480 cells, treatment with 7.5 µM MS13 for 48 h demonstrated an approximately 3-fold increase in caspase-3 activity relative to the untreated cells (*p* ≤ 0.05). The treatment with 15 µM MS13 for 48 and 72 h showed increased caspase-3 activity approximately by 4-fold compared to the untreated cells (*p* ≤ 0.01). Interestingly, caspase-3 activity was highest in SW620 cells when incubated for 24 h with both MS13 dosages of 5.7 µM (5-fold change; *p* ≤ 0.01) and 11.4 µM (8-fold change; *p* ≤ 0.001). In contrast, decreased caspase-3 activity was observed in SW620 with increased dosage and incubation time. 

### 2.6. MS13 Decreased B-cell Lymphoma (Bcl-2) Protein Concentrationin Colon Cancer 

**Figure 8:**
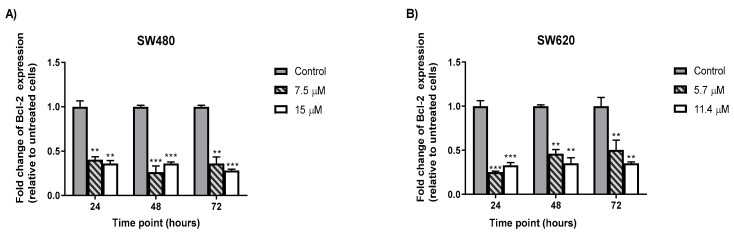
MS13 reduces BCl-2 expression level at different time points in SW480 (**A**) and SW620 cells (**B**). Experiments were performed in duplicates and compared between two independent experiments. Asterisk indicate statistically significant (* for *p* ≤ 0.05, ** for *p* ≤ 0.01, *** for *p* ≤ 0.001 and **** for *p* ≤ 0.0001) differences between data sets for each treatment dose. Error bars indicate mean ± SEM.

Significant reduction of Bcl-2 protein concentration was observed at all time points (24, 48 and 72 h) of all dosages of MS13 treatment in SW480 and SW620 compared to the untreated cells. It was also observed that the reduction of Bcl-2 protein expression was almost similar across each time point, although differ in MS13 dosage between both cell lines (SW480 and SW620)(Figure 8). 

## 3. Discussion 

Curcumin, a naturally occurring phenolic compound is widely reported to possess anti-cancer activities against many types of cancer [57,58,59,60] and may synergically improve CRC chemo-drug efficacy [61]. However, due to its poor absorption and rapid metabolism, efforts were made to improve its chemical properties by complexing it with lipids or modification of its molecular structure [42,62,63,64]. In the present study, MS13, a curcumin analogue synthesized by modification of curcumin’s phenolic rings and β-diketone moiety was observed to inhibit SW480 and SW620 colon cancer cell growth. MS13 was shown to be more cytotoxic and improved in growth inhibitory effect towards SW480 and SW620 compared to the parent compound, curcumin, in a dose-dependent manner. Both MS13 treated cancer cells exhibited approximately about 4-times more effective than curcumin in reducing cell viability by 50% based on the EC_50_ values (Table 1). Strong inhibition of MS13 was observed on colon cancer treated cells at 12.5 µM with approximately 10% viability (Figure 2). Previous studies by Citalingam et al., (2015) [47] and Paulraj et al., (2015) [49] also displayed greater inhibition of MS13 on prostate and cervical cancer cell viability. Other DAPs with a similar structure as MS13 such as FLLL-11 and GO-Y022 were observed to inhibit cell viability of colon (HCT116, HT29, and SW480) [65], pancreatic (PANC-1, BXPC-3, MIA-PACA-2, ASPC-1, and HPAC) [65] and gastric (KATO III, GCIY, H-111-TC, and SH-10-TC) cancer cell lines at a much lower dose than curcumin [66]. Besides, a product of curcumin pyrolysis during cooking and heating, known as “deketene curcumin” was observed to share similar structure as MS13, also demonstrated significant reduction of cell viability in melanoma cells compared to curcumin [67]. As such, this highlights the potency of curcumin analogue with 5-bis(4-hydroxy-3- methoxyphenyl)-1, 4-pentadiene-3-one structure in inhibiting cancer cell viability. 

Despite showing higher potency against both cancer cells, MS13 remains less cytotoxic towards normal cell CCD-18co and WRL-68 as supported by the selectivity index (SX) value (Table 2). MS13 appeared to be more selective towards cancer cells than normal cells. It is noted that MS13 at higher doses of 25 µM onwards may cause toxicity to both normal and colon cancer cells while doses as low as 5.7 µM (based on the EC_50_ value of MS13) are selectively toxic to the colon cancer cells. Cen et al. (2009) [45] observed that DAP-FLLL-11 was less cytotoxic as curcumin to human normal cell lines of lung fibroblast (WI-38), bladder smooth muscle and mammary epithelial (non-malignant MCF-10A). Its dose ranging from 1µM to 5µM has been reported to selectively toxic and increase in sensitivity towards colon cancer cells even up to 223-fold than normal cells [45]. It was also demonstrated that SW620 cells seems to be more sensitive to MS13 treatment compared to SW480 cells. It may be suggested that the sensitivity of MS13 towards SW620 might be due to the expression levels of Hsp70 (heat shock protein-70) and Hsp27 (heat shock protein-27). Rashmi et al., (2003) [68] reported that SW480 expressed higher level of Hsp70 and Hsp27 than SW620 cells. Earlier studies on tumor cell lines with higher expression of Hsp70 showed resistant towards curcumin treatment [69]. 

The effective dose of MS13 on cell growth suppression seems to be cell-specific, and different cancer cell lines require different doses, while inhibition of cell proliferation rate by MS13 treatment at 24, 48, and 72 h was in a time-dependent manner. To the best of our knowledge, it is believed that this was the first study that reported anti-proliferative activities of MS13 on colon cancer cells ranging from a low dosage of 1.6 µM to a higher dosage of 100µM at 24, 48 and 72 h (Figure 4A). MS13 treatment at 12.5 µM and 3.1 µM onwards significantly reduce cell viability of SW480 and SW620 colon cancer cells respectively and are more prominent with the increase in the duration of the incubation times. Significantly higher anti-proliferative activity was noted in both colon cancer cell lines (SW480 and SW620) following MS13 treatment for 48 and 72 h compared to 24 h. A significant declining pattern of cell viability was observed at 6.3 µM onwards following MS13 treatment for 48 and 72 h in SW480 primary colon cancer cells. Rapid decline of cell viability was also noted upon MS13 treatment of 3.1 µM on SW620 metastatic colon cancer cells at all time points. Curcumin however exerts anti-proliferative effect at a higher dosage of 50 µM and 25 µM onwards on SW480 and SW620 colon cancer cells respectively at all time points compared to the MS13 (Figure 4B). 

Several factors were suggested to explain the increase of cytotoxicity activity demonstrated by the MS13 compared to curcumin against cancer cells which includes the removal of β-diketone [44], substituents of 3′,4′-dimethoxy or 3′-methoxy-4′-hydroxy on the phenyl rings [50] and reduction of the electron-donating ability of the OH at the 4′ position [44]. MS13 is a mono ketone derivative carrying α and β unsaturated ketone meioty. It was suggested that the removal of this β-diketone may increase the growth-suppressive activities of MS13 as demonstrated by the growth-suppressive activities of other curcumin analogues containing a 5-carbon enone spacer without β-diketone against prostate cancer cells [34,44,70]. The α,β-unsaturated ketone may act as a Michael acceptor for nucleophilic groups thus improved the cytotoxic potential of the compound [33]. It has also been described that methoxy groups in curcumin are vital for its anti-proliferative activity [71]. Substitution of 4-hydroxy-3-methoxy aromatic rings and lacking only one of the carbonyls and the central methylene carbon joining by the five carbon spacers may reduce the electron-donating ability of the OH [44,45,72,73], thus increase cytotoxic effect against prostate (LNCaP and PC-3) [44,50,74], nasopharyngeal (CNE) [44], colon (LS174T) [44,75] and breast [74] cancer cell lines. 

It is well understood that one of the cytotoxicity effects and anti-proliferative activities of a tested cytotoxic compound might be associated with apoptosis. The FLLL-11 was found not to induce PARP cleavage in normal colonic smooth muscle cells which was the opposite in the human colorectal cancer cell lines (HCT-116, SW480, and HT-29) [45]. Cleavage of PARP has been noted as one of the signs of an apoptosis indicator [76,77]. Other factors have also been suggested which include the involvement of reactive oxygen species (ROS). Moderate levels of ROS may cause inflammation, cell damage, DNA mutation, but prolong exposure may lead to carcinogenesis [78], while excessive of ROS has been reported to promote apoptosis. Such examples are seen in 5-fluorouracil and vitamin C which at high doses increase ROS formation leading to apoptosis in CRC [79,80]. Dai F. et al., (2015) [81] reported that DAP bearing two ortho substituents on the aromatic rings referred as A1 (1,5-bis (2-trifluoromethyphenyl)-1, 4-pentadien-3-one), B1 (1,5-bis(2- hydroxyphenyl)- 1,4-pentadien-3-one) and C1 (1,5-bis (2-methoxyphenyl)-1, 4-pentadien-3-one) may increase the cytotoxic and proapoptotic activities of A549 lung cancer cells. The increased cytotoxic and proapoptotic activities were described via Michael acceptor unit which is dependent on prooxidant-mediated mechanism by converting the antioxidant enzyme-TrxR into a ROS promoter. In addition, its parent compound curcumin was also noted to induce apoptosis through generation of ROS in malignant cells but not in normal cells [82,83,84,85]. Apoptosis induction by curcumin has been reported to be selective of cell type and may rely on the ability of one cell to produce superoxide radical and the Hsp70 expression level [69,86,87]. Hence, therefore these might be the reasons explaining the sensitivity and selectivity of MS13 towards colon cancer cells rather than normal cells. The selectivity and sensitivity of DAPs towards cancer cells, and the dosages that vary among the cancer cells remain unclear and are not fully understood. To the best of our knowledge, the majority of DAPs demonstrated poor radical scavenging activity although it is common that the presence of hydroxyl group on phenyl ring is important in scavenging free radicals [88,89]. Previous studies showed that antioxidants may serve as cancer promoting agents [90,91]. 

Abruption in apoptosis pathways are commonly associated with CRC [16,92,93]. Dysregulation in apoptosis pathways may include alteration of genes responsible in apoptosis modulation such as *KRAS* and *TP53*. It has been reported that about 30% of CRC cases were linked to *KRAS* oncogene mutations [94] and about 70% were linked to the p53 mutations [95,96,97], where involvement includes cell survival, proliferation, and dysregulation of apoptosis [94,98,99,100]. In a normal colonic epithelium, a high number of apoptotic cells may be found at the top of the colon crypt. Disruption in apoptosis which is highly regulated and conserved programmed cell death may lead to the imbalance of colonic epithelia homeostasis [16,101,102]. In this regard, inhibition of cell proliferation and apoptosis induction are also one of the strategies for cancer treatment [103,104]. It is noted that the maintenance of the tumor phenotypes highly dependent on the alteration and disruption of these components [105]. The efficacy of cancer drugs such as Cetuximab, panitumumab, and Bevacizumab for metastatic CRC have been reported to correlate with the induction of apoptosis in cancer cells, inhibition of cell proliferation thus increases patient survival [106,107]. Since apoptosis may be stimulated by cytotoxic stress, development of effective anti-cancer therapeutic agents with such approach may be beneficial [80]. However, it was noted that not only ROS but drugs [80], physical agents (temperature, pH or osmolarity), chemicals (anti-cancer agents) and intracellular stresses may activate pro-apoptotic secondary messengers such as calcium, ceramide, cAMP and iron which then trigger cell death [108,109] via the mitochondria-mediated pathway or other appropriate cell death signaling pathways [108,109].

Therefore, the cause of cell death in correlation to the reduced number of viable colon cancer cells upon MS13 treatment was investigated via apoptosis by quantification of apoptotic cells, measuring caspase-3 activity and Bcl-2 protein level at dosages of EC_50_ and 2XEC_50_ on SW480 and SW620 cells for 24, 48, and 72 h. MS13 treatment on SW480 and SW620 cells has significantly increased the number of apoptotic cells. The cells undergone such changes were characterized by the visibly distinguished morphological changes of the Annexin-PI [56] as described earlier. In SW480 colon cancer cells, early apoptotic cells were observed following 7.5 µM of MS13 treatment at 24, 48 and 72 h, while an increased combination of early and late apoptotic cells was noted when treated with 15 µM for all time points. MS13 induced highest apoptosis activity which represent approximately 79% of the cell population following treatment at 72 h. Incubation for 24 h at 15 µM induced late apoptosis, and the presence of necrotic cells remained approximately less than 11% were noticed at 72 h upon MS13 treatment of 7.5 µM and 15 µM. However, in SW620 cells significant increase in early apoptotic cells were observed following MS13 treatment of 5.7 µM and 11.4 µM for 24 h. Prolonged treatment of MS13 for 48 h and 72 h induce late apoptosis as seen by the increased number of yellow-orange and orange cells. The number of late apoptotic cells were proportionate with the increased of MS13 dosage and incubation time. It was noted that MS13 increased apoptosis induction by approximately 38% and 75% of the cell population when treated with 5.7 µM and 11.4 µM for 72 h, respectively. As such, the morphological assessment of MS13 treated cells, revealed the ability of cell death induction via apoptosis in a dose- and time- dependent manner. 

The induction of apoptosis and its downstream signaling also includes caspase-3 and Bcl-2 [110,111,112]. Caspase-3 is one of the important enzymes involved in apoptosis. It serves as executioner caspase that cleaves substrates including PARP and activation cascade of caspases responsible for apoptosis execution. Caspase-3, which serves as caspase executioner, is a member of the endoproteases family that regulates apoptosis signaling networks, and as shown in Figure 7, the treatment of colon cancer cells with MS13 resulted in increased levels of caspase-3 activity. It was observed that caspase-3 activity in both cell lines were dose and time point specific. SW480 has highest caspase-3 activity at 48 h upon 7.5 µM of MS13 treatment, while SW620 has the highest caspase-3 activity at 24 h upon 11.4 µM of MS13 treatment. On the other hand, decrease of Bcl-2 protein level was observed in MS13-treated colon cancer cells (Figure 8). Bcl-2 is an anti-apoptotic protein that serves as a negative regulator of apoptosis. The decrease in Bcl-2 levels leads to cell death via apoptosis [113]. 

In this study, it was suggested that increased in caspase-3 activity and suppression of Bcl-2 may contribute to the MS13-induced apoptosis, evidenced by morphological staining. Shrinkage of cells, blistering and membrane blebbing were the morphological changes observed in SW480 and SW620-MS13 treated cells indicating apoptosis (Figure 5A,B). FLLL-11 which is identical to MS13 was believed to inhibit cell viability, proliferation and induce apoptosis via caspase 3 in human colorectal cancer cell lines [45]. It was reported that DAPs demonstrated anti-cancer effect through induction of apoptosis in various human colon cancer cell lines involving activation of caspase-3 [28,39,114,115] and down-regulation of anti-apoptotic Bcl-2 protein [116,117] even at a very low concentration between the range of 2.5–6.0 µM [45,117]. Another DAP, 1,5-bis (2-metoxyphenyl)-1,4-pentadiene-3-one (B63) was shown to induce apoptosis in human colon cancer cells via the mitochondrial apoptotic pathway which includes down-regulation of mitochondrial complexes, up-regulation of pro-apoptotic proteins-Bad and Bim, activation of caspase-3, PARP cleavage and cytochrome c release [119]. Induction of apoptosis by B63 is dependent on the accumulation of ROS leading to ER stress which then caused mitochondrial disfunction [118]. The down-regulation of the mitochondrial complexes (I-IV) [118] and inhibition of signal transducer and activator of transcription 3 (STAT3) were also observed in in vivo study of a tumor xenograft derived from athymic nude mice which exhibited greater anti-tumor effect compared to curcumin [119,120]. In addition, Yoshida et al. (2018) reported that GO-Y022, a DAP with a similar structure to MS13, was able to suppress gastric tumor growth in transgenic mice (K19-Wnt/C2mE), inhibit phosphorylation of STAT3 without hepatic and renal toxicity, retain localized in tumor areas, as well as exhibit better pharmacokinetic profiles than curcumin [44] when ingested orally [66].

Although in the present study the induction of apoptosis by MS13 is suggested via increased caspase-3 activity and down-regulation of Bcl-2 expression, several DAPs were shown to induce various apoptotic pathways in colon cancer cells which include alter expression of pro-apoptotic (Bax and Bad) and pro-survival proteins (Bcl-2 and Bcl-xL), ROS-ER-stress pathway, inhibition of STAT3 phosphorylation [116,119,120,121,122], degradation of β-catenin and down-regulation of Ki-ras [123]. The degradation of β-catenin and down-regulation of Ki-ras however, was prohibited in the presence of caspase-3 inhibitor [123]. Therefore, in this study, MS13 might promote colon cancer cell apoptosis through the activation of caspase-3 and decrease of Bcl-2 protein level.

## 4. Materials and Methods 

### 4.1. Colon Cancer Cell Lines

Normal human colon (CCD-18co, ATTC^®^ CRL-1459TM) and hepatic (WRL-68, ATCC^®^ CL-48TM) cell line, primary (SW480, ATCC^®^ CCL-228™) and metastatic (SW620, ATCC^®^ CCL-227™) human colon cancer cell lines were purchased from the American Type Culture Collection (ATCC, Manassas, VA, USA). CCD-18co and WRL-68 cells were grown in Eagle’s minimum essential medium (EMEM) (Corning Life Sciences, Corning, NY, USA); and stored in a humidified 37 °C incubator with 5% CO_2_. Both colon cancer cell lines were grown in Leibovitz L-15 medium (Corning Life Sciences, Corning, NY, USA); and stored at 37 °C in a humidified incubator without CO_2_. All media were supplemented with 10% fetal bovine serum (FBS, Gibco, Grand Island, NY, USA) and 1% penicillin (100 U/mL)/ streptomycin (100 ug/mL) (Gibco). Cells were regularly monitored to ensure a normal and consistent morphology without contaminants. Cells were maintained by proper aseptic techniques and left to grow until it reached a confluency of 80%–90%.

### 4.2. Preparation of Curcumin and Curcumin Analogue, MS13

Curcumin analogue-MS13 (1,5-bis(4-hydroxy-3-methoxyphenyl)-1,4-pentadiene-3-one) was synthesized by coupling the appropriate aromatic aldehyde with acetone and cyclohexanone under base-catalyzed aldol condensation conditions, using a 1:2 ratio of ketone to aldehyde [88]. The characterization of the analogue was based on the analysis of its spectroscopic data and comparison of this data with the related compounds. Curcumin was purchased from Sigma (Sigma Aldrich, St. Louis, MA, USA). 50 mM concentration of MS13 and curcumin stock solutions were prepared in DMSO (DMSO, Molecular Biology Grade, Sigma Aldrich, St. Louis, MO, USA). 

### 4.3. Cell Viability and Anti-Proliferative Assays

The cells were seeded in a 96-well flat-bottomed microtiter plate (Nunc, Roskilde, Denmark) at a density of 7 × 10^4^ cells/mL (7 × 10^3^ cells/ 0.1 mL/well) together with appropriate culture media in triplicates. Cells were left to adhere to the bottom of the wells for 24 h in a humidified incubator at 37 °C, before replaced with fresh media containing MS13 or curcumin ranging from 1.6 µM–100 µM by a two-fold serial dilution. The compounds were previously prepared as described by Paulraj et al., 2015 [49] and Citalingam et al., 2015 [47]. Cells were treated for 72 h for dose-dependent cytotoxicity assays whereas 24, 48, and 72 h for anti-proliferative assays (time-dependent). Control cells were treated with media contained 0.2% dimethyl sulfoxide (DMSO, Molecular Biology Grade, Sigma Aldrich, St. Louis, MO, USA). To evaluate the cell viability and anti-proliferative activity upon treatment, 3-(4,5-dimethylthiazol-2-yl)-2,5-diphenyl tetrazolium bromide (MTT) assay was performed by replacing the media in each well with 100 mL complete medium of 0.5 mg/mL MTT solution and incubated for 4 h at 37 °C. The MTT was again replaced with 100 mL of DMSO to dissolve the formazan crystals. The absorbance of the formazan was read at 570/650 nm wavelengths using a microplate spectrophotometer (BioTekTM EONTM Microplate Spectrophotometers, Fisher Scientific, Suwanee, GA, USA). The calculation of cell viability was as follows:

(1) $$\mathrm{Cell}\;\mathrm{Viability}\%=\frac{\mathrm{Average}\;\mathrm{Absorbance}\;\mathrm{of}\;\mathrm{Treated}\;\mathrm{Cells}}{\mathrm{Average}\;\mathrm{Absorbance}\;\mathrm{of}\;\mathrm{Untreated}\;\mathrm{Cells}\;\left(\mathrm{Control}\right)}$$

EC_50_ values were generated from GraphPad Prism 7 software (Graphpad Software, La Jolla, CA, USA) with non-linear regression curve fits of the data. The EC_50_ represents the concentration of the compound required to reduce cell viability by at least 50% of the cell population, averaged from 3 independent experiments. The selectivity index (SX) values of the compounds were calculated using the equation below, which was adapted from Popiolkiewicz et al., 2005 [124].

(2) $$\mathrm{Selectivity}\;\mathrm{Index}\;\left(\mathrm{SX}\right)=\;\frac{{\mathrm{EC}}_{50}\;\mathrm{normal}\;\mathrm{colon}\;\mathrm{cell}\;\mathrm{line}\;}{{\mathrm{EC}}_{50}\;\mathrm{colon}\;\mathrm{cancer}\;\mathrm{cell}\;\mathrm{line}}\times 100$$

The SX value above 100 indicates a high cytotoxic selectivity of the tested compound against cancerous cells compared to the non-cancerous cells. 

### 4.4. Morphological Analysis of Apoptotic Cells by Annexin V

The cell morphology changes were assessed by using the Annexin V Apoptosis Detection Kit (Raybiotech Inc., Peachtree Corners, GA, USA) according to manufacturer’s instruction. Briefly, colon cancer cells were plated in 6-well plates at a concentration of 7 × 10^6^/mL/well and left for 24 h before exposed to treatment for 24, 48 and 72 h. Harvested cells were resuspended in the binding buffer. Annexin V-FITC and PI were added to the binding buffer and incubated at room temperature for 10 min in the dark. 10 µL of cell suspension was pipetted onto a clean microscope slide. Fluorescent detection was observed under a fluorescence microscope using the fluorescein filter (BX41, Olympus, Melville, NY, USA) using a dual filter set for FITC (green) and rhodamine (red). Apoptotic cells have bound Annexin V-FITC represented by the green plasma membrane or red staining (PI) throughout the nucleus accompanied with halo of green staining (Annexin) on the plasma, or cells stained green to yellow with loss of membrane integrity and/or condensed or fragmented yellow-orange or orange chromatin. Necrotic cells showed pale orange or uniformly red in appearance [54,55,56]. 

To quantify the apoptotic and necrotic cells undergoing MS13 treatment, a minimum of 200 cells were counted per sample, and percentage of cells from each population (viable, apoptotic, and necrotic cells) was calculated [54,125] as the equation below:

(3) $$\%\;of\;cells=\frac{number\;of\;viable\;or\;apoptotic\;or\;necrotic\;cells}{200\;cells}\times 100$$

### 4.5. Determination of Caspase-3 Activity

Caspase-3 activity was determined using the Caspase-3 Colorimetric Assay Kit (Raybiotech Inc., Peachtree Corners, GA, USA), according to the manufacturer’s instruction. The colon cancer cells were seeded and undergone treatment for 24, 48, and 72 h at different concentrations (EC_50_, 2XEC_50_) before suspended with lysis buffer. The protein lysate concentration was then determined using Pierce BCA Protein Assay Kit (Thermo Fisher Scientific, Waltham, MA, USA) following the manufacturer’s protocol. Caspase-3 activity was measured by the intensity of yellow-colored of the pNA molecule at 405 nm by using a microplate spectrophotometer (BioTek^TM^ EON^TM^ Microplate Spectrophotometers, Fischer Scientific, Suwanee, GA, USA). The caspase-3 activity was presented in fold-change of absorbance from treated cells against absorbance from untreated cells as follows:

(4) $$Fold\;change\;of\;caspase-3\;activity=\;\frac{Absorbance\;of\;MS13\;treated\;cells}{Absorbance\;of\;untreated\;cells\;\left(control\right)}$$

### 4.6. Determination of Bcl-2 Cellular Protein Concentration

Bcl-2 protein concentration was determined using the Bcl-2 ELISA kit (Invitrogen, Thermofisher Scientific, Waltham, MA, USA) according to the manufacturer’s instruction. The colon cancer cells were plated and treated for 24, 48, and 72 h at different concentrations (EC_50_, 2XEC_50_). The Bcl-2 concentration was according to the color intensity measured at 450 nm by using a microplate spectrophotometer (BioTek™ EON™ Microplate Spectrophotometers, Fisher Scientific, Suwanee, GA, USA). Data was presented in fold-change of absorbance from treated cells against absorbance from untreated cells as follows:

(5) $$Fold\;change\;of\;Bcl-2=\;\frac{Absorbance\;of\;MS13\;treated\;cells}{Absorbance\;of\;untreated\;cells\;\left(control\right)}$$

### 4.7. Statistical Analysis 

The results were presented as the mean ± standard deviation (SD). All samples were measured in triplicates (n = 3) from each independent experiment. Comparison between sets of data was performed using one-way analysis of variance (ANOVA) followed by Dunnett’s multiple group comparison tests. Statistically significant differences between the groups were accepted at *p* ≤ 0.05. All statistical analyses were performed using GraphPad Prism Version 7.0 (GraphPad Software, Inc., La Jolla, CA, USA).

## 5. Conclusions

In summary, it can be concluded that 1,5-bis(4-hydroxy-3-methoxyphenyl)-1,4 -penta-diene-3-one (MS13) is more potent compared to its native compound curcumin as shown by the low EC_50_ value and able to suppress colon cancer cell growth by inhibiting cell proliferation and induces apoptosis. MS13 demonstrated significant cytotoxic and anti-proliferative effects in a time and dose-dependent manner. MS13 induced apoptosis as shown by typical apoptotic morphological changes, and significant increase of caspase-3 activity and reduction of the Bcl-2 levels. Therefore, it is suggested that MS13 may possess anti-cancer activity, and the findings lays foundation for further investigation to fully elucidate the mechanism of action of MS13 as potential anti-cancer agent. 

## Figures and Tables

**Table 1:** EC_50_ values of MS13 and curcumin on colon cancer (SE480 and SW620) and normal (WRL-68 and CCD-18co) cells.

Cell Line	EC_50_ Values (µM) of Compounds	
	MS13	* Curcumin
SW480	7.5 ± 2.8	30.6 ± 1.4
SW620	5.7 ± 2.4	26.8 ± 2.1
WRL-68	8.8 ± 0.6	30.3 ± 1.5
CCD-18co	9.8 ± 0.7	28.0 ± 1.8

* Curcumin was used as a positive control. The results are shown as mean ± standard deviation (SD) from three independent experiments.

**Table 2:** Selective index (SX) values of MS13 and curcumin treated colon cancer (SW480 and SW620) and normal (WRL-68 and CCD18-co) cells.

Compound	WRL-68 (Normal Human Epithelial Hepatocytes)		CCD-18co (Normal Human Colon Fibroblast)	
	SW480	SW620	SW480	SW620
MS13	117.3	154.4	130.7	171.9
Curcumin	99	113.1	91.5	104.5

*SX* values > 100 indicates that the cytotoxic effect of the tested compound is greater towards cancer.
